# Lack of Immunotherapy as the Only Predictor of Secondary Generalization in Very-Late-Onset Myasthenia Gravis With Pure Ocular Onset

**DOI:** 10.3389/fneur.2022.857402

**Published:** 2022-04-25

**Authors:** Sijia Zhao, Xu Yan, Jiaqi Ding, Kaixi Ren, Shuyu Sun, Jiarui Lu, Chao Zhang, Kai Zhang, Zhuyi Li, Jun Guo

**Affiliations:** ^1^Department of Neurology, Tangdu Hospital, Air Force Medical University, Xi'an, China; ^2^Department of Neurology, Suide County Hospital, Yulin, China; ^3^Department of Intensive Care Unit, Xi'an, Hospital, Xi'an, China

**Keywords:** very-late-onset, myasthenia gravis, predictor, immunotherapy, generalization

## Abstract

During the past two decades, an increasing number of patients with very-late-onset myasthenia gravis (v-LOMG) with an onset age of 65 years or older have been identified. However, few studies explore the predictors of secondary generalization in patients with v-LOMG with pure ocular onset. In this retrospective cohort study, 69 patients with v-LOMG were divided into ocular MG (OMG) and generalized MG (GMG), and the clinical characteristics and outcomes were compared. Cox regression analysis was performed to explore the predictors of generalization. The average onset age of the study population was 73.1 ± 4.2 years and the median disease duration was 48.0 months (interquartile range, 32.5–64.5 months). Serum acetylcholine receptor (AChR) antibody was detected in up to 86% of patients and concomitant diseases in approximately half of the patients. Male predominance was seen in OMG group while female predominance in GMG group (*p* = 0.043). Patients with OMG showed a lower positive rate of repetitive nerve stimulation (RNS) than those with GMG (*p* = 0.014), and favorable outcomes were obtained in more patients with OMG than those with GMG (*p* < 0.001). Of the 51 patients with pure ocular onset, 25 (49.0%) underwent secondary generalization. A higher probability of generalization was found in patients with positive RNS results and without immunotherapy (*p* = 0.018 and <0.001). Upon Cox regression analysis, immunotherapy was negatively associated with secondary generalization [HR (hazard ratio) 0.077, 95%CI [0.024–0.247], *p* < 0.001]. Altogether, compared to the patients with very-late-onset GMG, the counterparts with OMG exhibit a significantly higher female predominance and a lower positive rate of RNS tests, especially on facial and accessory nerves. Lack of immunotherapy is the only predictor of secondary generalization in those with pure ocular onset.

## Introduction

Myasthenia gravis (MG) is an organ-specific autoimmune disease characterized by the presence of pathogenic antibodies mainly targeting acetylcholine receptors (AChRs) located at the neuromuscular junctions, leading to fluctuating and fatigable weakness of skeletal muscles. With the onset age of 50 years as the boundary, MG is categorized into early-onset MG (EOMG) and late-onset MG (LOMG) with different demographic and clinical profiles, indicating the requirement for classification of this disease (1–3). In recent decades, owing to the extensive application of diagnostic testing and gradual improvement in living conditions, an increasing number of patients with very-late-onset MG (v-LOMG) with an onset age of 65 years or older have been identified (4), and patients with elderly onset appear to exhibit unique demographic and clinical characteristics from EOMG and LOMG (3, 5–8). It is noted that older age is more likely to be accompanied by comorbidities, more fragility to medication side effects, and aging-related changes of immune system (9, 10), which may influence the clinical phenotype. Hence, it is of great significance to further outline the picture of the subgroup with v-LOMG.

Based on the muscles involved, MG can be divided into ocular MG (OMG) and generalized MG (GMG). To date, secondary generalization in patients with pure ocular onset has been identified as a well-known hallmark of MG. Once generalized symptoms develop, the patient's clinical status would become worse and might be associated with a poorer prognosis. Although studies have indicated the importance of considering factors including onset age, AChR antibody status, thymoma, and immunotherapy as predictors of secondary generalization in patients with MG of different ages (11–15), risk factors for generalization in the population with v-LOMG have not been established as far. Herein, we conducted a retrospective cohort study enrolling patients with v-LOMG from a tertiary hospital in Northwest China to outline the clinical picture of v-LOMG in the Han Chinese population and explore the predictors of secondary generalization in this unique subgroup.

## Methods

### Patient Enrollment and Data Collection

**Figure 1:**
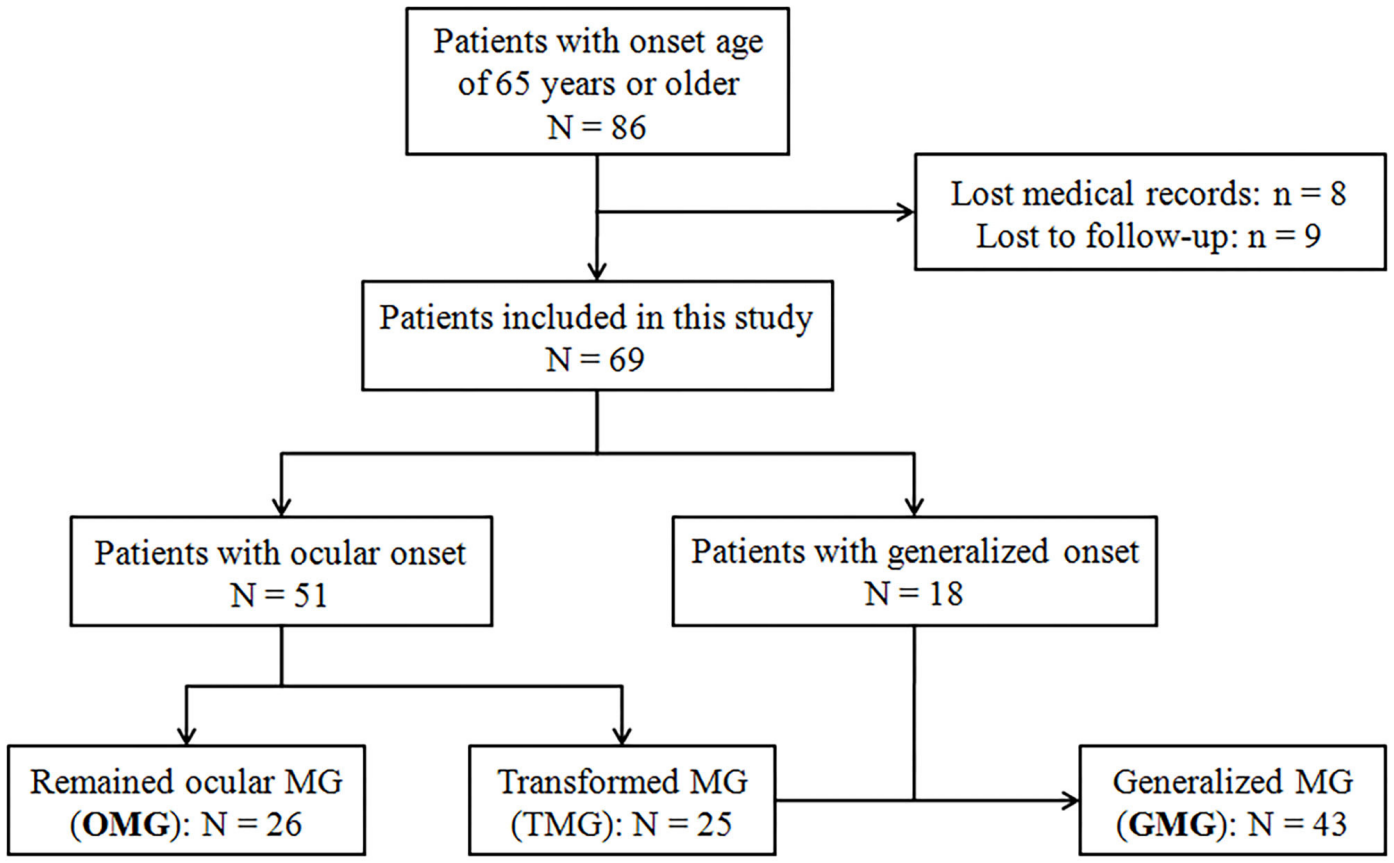
Flowchart of the enrollment of the patient with v-LOMG in this study.

All patients with MG with an onset age of 65 years or older at outpatient and inpatient units of the Department of Neurology, Tangdu Hospital, between January 2017 and July 2020 were recruited in this study. The patients with complete medical and follow-up records were eventually enrolled after the written informed consent was obtained. Figure 1 showed the flowchart of patient enrollment and grouping. A definite MG diagnosis was made based on the clinical symptoms of fluctuating, fatigable skeletal muscles weakness, and the evidence of at least one of the following items: (1) unequivocal response to cholinesterase inhibitor; (2) positive response to repetitive nerve stimulation (RNS) with amplitude decrement of >10% in compound muscle action potential; or (3) seropositivity for AChR antibody measured by radioimmunoprecipitation assay. The last follow-up visit was performed in August 2021 to ensure the disease duration of all the enrolled patients was 2 years or longer. Patients with confined ocular involvement till the last follow-up were defined as pure OMG, whereas those with pure ocular onset but undergoing secondary generalization were defined as transformed MG (TMG), and GMG consisted of TMG and those with generalized onset. Demographic data including gender, onset age, disease duration (from onset to the last follow-up), initial symptoms, AChR antibody status, RNS test results, thymic abnormalities on CT scan, and concomitant diseases at the initial contact were collected and then compared between OMG and GMG groups. Immunotherapy regimens in the course of disease were collected and divided into 3 groups: steroids only, steroids plus other immunosuppressants (IS), and IS only. In this study, IS included azathioprine, tacrolimus, mycophenolate mofetil, and intravenous immunoglobulin (IVIg). Clinical outcome was evaluated at the last follow-up (August 2021) by Myasthenia Gravis Foundation of America Post-Intervention Status (MGFA-PIS) and the achievement of minimal manifestations (MM) or better [including complete stable remission (CSR) and pharmacologic remission (PR)] was defined as favorable outcomes. A status of improved (I) was categorized as an intermediate outcome. Unchanged (U), worse (W), and exacerbation (E) were classified as unfavorable outcomes. Died (D) of MG was defined as a poor outcome. This study was approved by the Ethics Committees of Tangdu Hospital, Air Force Medical University (approval number: TDLL-KY-202105-04). Written informed consent was waived in accordance with the institutional requirements because of the retrospective nature of this study. As an alternative, oral informed consent to participate in this study was obtained from all the patients.

### Statistical Analysis

Categorical variables were presented as number with percentage and numerical variables as mean with standard deviation (SD) or median with interquartile range (IQR). Statistical analysis was performed by the SPSS 23.0 software (SPSS Inc., Chicago, IL, USA). Intergroup differences of categorical variables were evaluated by χ^2^ test and Fisher's exact test when necessary, and those of continuous variables were compared by Student's *t*-test or Mann–Whitney U test. The probability of secondary generalization was presented using the Kaplan–Meier method and analyzed with the log-rank test. Cox regression analysis was performed on variables of interest to identify the predictors of secondary generalization. Hazard ratio (HR) with 95% confidence intervals (CI) was calculated. A value of *p* < 0.05 was considered statistically significant in all analyses.

## Results

### General Information

As shown in Figure 1, 69 of the initially identified 86 patients entered into the final analysis. In general, this population with v-LOMG exhibited unique features such as high AChR antibody seropositivity in up to 86% of patients and predominant concomitant diseases in approximately 50% of patients (Table 1).

**Table 1:** Demographic, clinical characteristics, and long-term outcomes of the patients with v-LOMG.

**Variables**	**MG**	**OMG**	**GMG**	***P*-value**
Gender	*N =* 69	*N =* 26	*N =* 43	
Male, *n* (%)	37 (53.6)	18 (69.2)	19 (44.2)	0.043
Female, *n* (%)	32 (46.4)	8 (30.8)	24 (55.8)	
Onset age (*y*), mean ± SD	73.1 ± 4.2	73.4 ± 3.9	72.9 ± 4.4	0.641
Disease duration (*m*), median (IQR)	48.0 (32.5–64.5)	49.5 (32.8–62.5)	45.0 (32.0–85.0)	0.985
Muscles initially involved	*N =* 69	*N =* 26	*N =* 43	
Ocular, *n* (%)	65 (94.2)	26 (100)	39 (90.7)	0.289^*^
Limbs, *n* (%)	7 (10.1)	NA	7 (16.3)	NA
Bulbar, *n* (%)	15 (21.7)	NA	15 (21.7)	NA
Axial muscles, *n* (%)	5 (7.2)	NA	5 (11.6)	NA
AChR antibody status	*N =* 63	*N =* 22	*N =* 41	
Positive, *n* (%)	54 (85.7)	20 (90.9)	34 (82.9)	0.476^*^
Negative, *n* (%)	9 (14.3)	2 (9.1)	7 (17.1)	
RNS test positive	*N =* 69	*N =* 26	*N =* 43	
Facial nerve, *n* (%)	29 (42.0)	6 (23.1)	23 (53.4)	0.013
Ulnar nerve, *n* (%)	6 (9.2)	1 (3.8)	5 (11.6)	0.398^*^
Axillary nerve, *n* (%)	30 (43.5)	8 (30.8)	22 (51.2)	0.098
Accessory nerve, *n* (%)	21 (30.4)	3 (11.5)	18 (41.9)	0.008
Any nerve, *n* (%)	42 (60.9)	11 (42.3)	31 (72.1)	0.014
Thymic abnormalities	*N =* 69	*N =* 26	*N =* 43	
Thymoma, *n* (%)	4 (5.8)	0 (0)	4 (9.3)	0.289^*^
Thymic hyperplasia, *n* (%)	18 (26.1)	8 (30.8)	10 (23.3)	0.491
Thymectomy, *n* (%)	6 (8.7)	0 (0)	6 (8.7)	0.067^*^
Concomitant diseases	*N =* 69	*N =* 26	*N =* 43	
Hypertension, *n* (%)	35 (50.7)	12 (46.2)	23 (53.5)	0.555
Diabetes, *n* (%)	20 (29.0)	8 (30.8)	12 (27.9)	0.800
Coronary heart disease, *n* (%)	13 (18.8)	6 (23.1)	7 (16.3)	0.535^*^
Cerebral infarction, *n* (%)	12 (17.4)	3 (11.5)	9 (20.0)	0.514^*^
Tumor, *n* (%)	4 (5.8)	1 (3.8)	3 (7.0)	1.000^*^
Immunotherapy	*N =* 63	*N =* 24	*N =* 39	
Steroids, *n* (%)	9 (13.0)	3 (11.5)	6 (14.0)	1.000^*^
Steroids + IS, *n* (%)	46 (66.7)	20 (76.9)	26 (60.5)	0.148
IS, *n* (%)	8 (11.6)	1 (3.8)	7 (16.3)	0.141^*^
Outcomes	*N =* 69	*N =* 26	*N =* 43	
Favorable, *n* (%)	45 (65.2)	24 (92.3)	21 (48.8)	<0.001
Intermediate, *n* (%)	12 (17.4)	0 (0)	12 (27.9)	0.002^*^
Unfavorable, *n* (%)	6 (8.7)	2 (7.7)	4 (9.3)	1.000^*^
Poor, *n* (%)	6 (8.7)	0 (0)	6 (14.0)	0.076^*^
Myasthenic crisis, *n* (%)	5 (7.2)	0 (0)	5 (11.6)	0.149^*^

*AChR, Acetylcholine receptor; GMG, generalized myasthenia gravis; IQR, interquartile range; MG, myasthenia gravis; m, month; NA, not applicable; OMG, ocular myasthenia gravis; RNS, repetitive nerve stimulation; IS, immunosuppressant; SD, standard deviation; v-LOMG, very-late-onset myasthenia gravis; y, year. Outcomes were evaluated by the Myasthenia Gravis Foundation of America Post-Intervention Status (MGFA-PIS). Favorable outcomes were defined as the achievement of minimal manifestations (MM) or better, including complete stable remission (CSR), pharmacologic remission (PR), and MM. An intermediate outcome was considered as a status of improved (I); unfavorable outcomes as unchanged (U), worse (W), and exacerbation (E); and a poor outcome as died (D) of MG. Intergroup difference of onset age was analyzed by Student's t-test and that of disease duration by Mann–Whitney U test. Otherwise, χ^2^ test was used to compare the intergroup differences*.

* *Fisher's exact test was performed. The values of p were drawn from the statistical analysis between the OMG and GMG groups*.

### Comparison of Demographic and Clinical Characteristics Between Different v-LOMG Subtypes

In total, 69 patients with v-LOMG (32 females, 37 males) had an average onset age of 73.1 ± 4.2 years and a median disease duration of 48.0 months (IQR, 32.5–64.5 months). Although no obvious gender difference (female-to-male ratio, 1:1.2) was present in the entire study population, a male predominance was prominent in OMG group in contrast to that in GMG group (*p* = 0.043). Upon RNS tests, the positive result of any nerve was recorded in more patients with GMG than counterparts with OMG (*p* = 0.014). Specifically, the positive rates of RNS tests on facial and accessory nerves were significantly higher in GMG group than those in OMG group (*p* = 0.013 and 0.008, respectively) (Table 1). Considering the possibility of secondary generalization in patients with v-LOMG with pure ocular onset, we further compared the baseline demographic and clinical characteristics between the remained patients with OMG and those with TMG. As shown in Table 2, no significant intergroup differences were observed except for significantly higher positive rate of RNS tests in TMG group (*p* = 0.032, compared with OMG group), in particular when repetitive stimulating facial and accessory nerves (*p* = 0.033 and 0.020, respectively).

**Table 2:** Baseline demographic and clinical data of patients with v-LOMG with pure ocular onset.

**Variables**	**Ocular-onset MG**	**OMG**	**TMG**	***P*-value**
Gender	*N =* 51	*N =* 26	*N =* 25	
Male, *n* (%)	31 (53.6)	18 (69.2)	13 (52.0)	0.208
Female, *n* (%)	32 (46.4)	8 (30.8)	12 (48.0)	
Onset age (y), mean ± SD	72.7 ± 4.3	73.4 ± 3.9	72.0 ± 4.5	0.223
Disease duration (m), median (IQR)	49.0 (33.0–66.0)	49.5 (32.8–62.5)	48.0 (44.5–94.5)	0.423
Initial clinical symptoms	*N =* 51	*N =* 26	*N =* 25	
Unilateral ptosis, *n* (%)	29 (56.9)	16 (61.5)	13 (52.0)	0.492
Bilateral ptosis, *n* (%)	2 (3.9)	0 (0)	2 (8.0)	0.235^*^
Unilateral ptosis with diplopia, *n* (%)	16 (31.4)	7 (26.9)	9 (36.0)	0.485
Bilateral ptosis with diplopia, *n* (%)	4 (7.8)	3 (11.5)	1 (4.0)	0.610^*^
AChR antibody status	*N =* 45	*N =* 22	*N =* 23	
Positive, *n* (%)	39 (86.7)	20 (90.9)	19 (82.6)	0.665^*^
Negative, *n* (%)	6 (13.3)	2 (9.1)	4 (17.4)	
RNS test positive	*N =* 51	*N =* 26	*N =* 25	
Facial nerve, *n* (%)	19 (37.3)	6 (23.1)	13 (52.0)	0.033
Ulnar nerve, *n* (%)	3 (5.9)	1 (3.8)	2 (8.0)	0.610^*^
Axillary nerve, *n* (%)	21 (41.2)	8 (30.8)	13 (52.0)	0.124
Accessory nerve, *n* (%)	13 (25.5)	3 (11.5)	10 (40.0)	0.020
Any nerve, *n* (%)	29 (56.9)	11 (42.3)	18 (72.0)	0.032
Thymic abnormalities	*N =* 51	*N =* 26	*N =* 25	
Thymoma, *n* (%)	2 (3.9)	0 (0)	2 (8.0)	0.235^*^
Thymic hyperplasia, *n* (%)	12 (26.1)	8 (30.8)	4 (16.0)	0.214
Thymectomy, *n* (%)	3 (5.9)	0 (0)	3 (12.0)	0.110
Concomitant diseases	*N =* 51	*N =* 26	*N =* 25	
Hypertension, *n* (%)	29 (56.9)	12 (46.2)	17 (68.0)	0.115
Diabetes, *n* (%)	15 (29.4)	8 (30.8)	7 (28.0)	0.828
Coronary heart disease, *n* (%)	10 (19.6)	6 (23.1)	4 (16.0)	0.726^*^
Cerebral infarction, *n* (%)	10 (19.6)	3 (11.5)	7 (28.0)	0.173^*^
Tumor, *n* (%)	4 (7.8)	1 (3.8)	3 (12.0)	0.350^*^
Immunotherapy	*N =* 47	*N =* 24	*N =* 23	
Time from onset to immunotherapy initiation (m), median (IQR)	5.0 (1.0–24.0)	4.0 (1.0–17.3)	7.0 (2.5–24.0)	0.156

*AChR, Acetylcholine receptor; IQR, interquartile range; m, month; OMG, ocular myasthenia gravis; RNS, repetitive nerve stimulation; SD, standard deviation; TMG, transformed myasthenia gravis; v-LOMG, very-late-onset myasthenia gravis; y, year. Intergroup difference of onset age was analyzed by Student's t-test and those of disease duration and time from onset to immunotherapy initiation by Mann–Whitney U test. Otherwise, χ^2^ test was used to compare the intergroup differences*.

* *Fisher's exact test was performed. The values of p were drawn from statistical analysis between the OMG and TMG groups*.

### Comparison of Long-Term Outcomes Between Different v-LOMG Phenotypes or Therapies

First, we compared the long-term outcomes between OMG and GMG groups irrespective of the therapies used. Favorable outcomes were obtained in a significantly higher proportion of patients in OMG group than in GMG group (92.3% vs. 48.8%, *p* < 0.001; Table 1). Then the study population was divided into two groups based on whether or not receiving immunotherapy in the course of disease and the duration of therapy (short-term, <6 months, vs. long-term, ≥6 months), respectively, and clinical outcomes were compared between groups. Meanwhile, the outcomes associated with different treatment strategies were analyzed. Although no significant differences in the proportion of patients with distinct outcomes were observed between different duration of therapy (Supplementary Table 1) and amongst distinct treatment strategies (Supplementary Table 2), immunotherapy indeed led to a significantly higher proportion of favorable outcome and a lower proportion of unfavorable outcome compared with those not receiving immunotherapy (71.4% vs. 0, *p* = 0.001 and 4.8% vs. 50%, *p* = 0.007; Supplementary Table 1). Moreover, patients receiving immunotherapy showed a lower proportion of developing myasthenic crisis than those not receiving immunotherapy (1.4% vs. 5.8%, *p* < 0.001).

### Secondary Generalization of v-LOMG With Pure Ocular Onset

**Figure 2:**
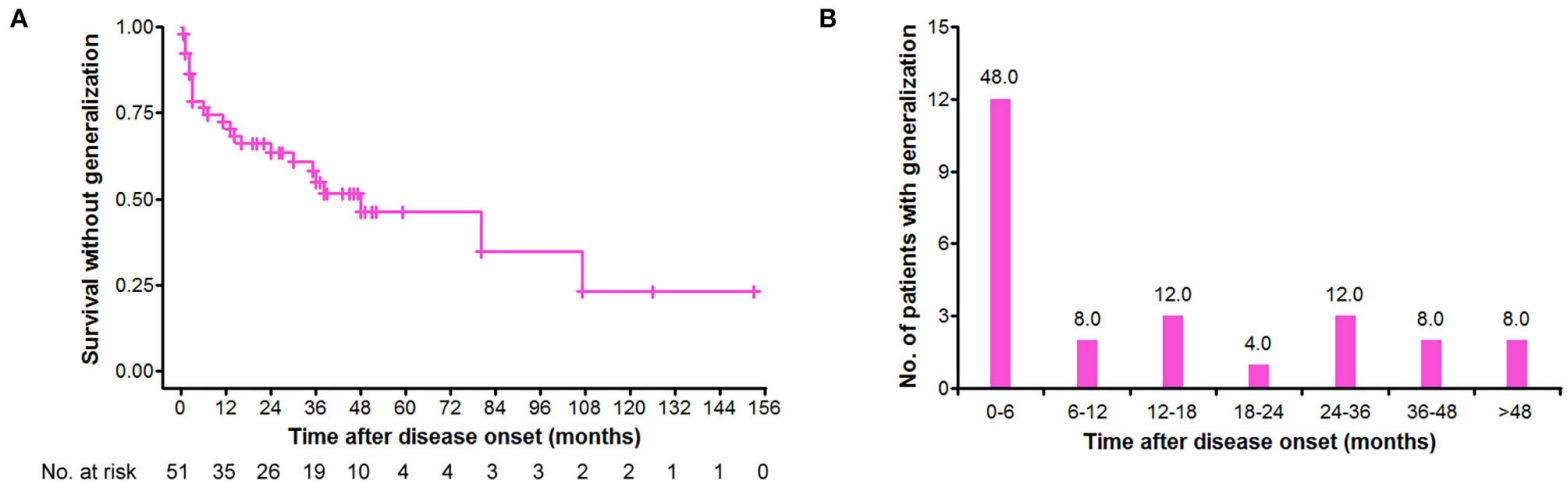
Distribution of secondary generalization in the patients with v-LOMG with pure ocular onset. **(A)** Kaplan–Meier curve depicting the cumulative survival without generalization over time (months). **(B)** Time distribution of generalization in the 25 patients with v-LOMG. *Y*-axis indicates the number of patients undergoing generalization with the percentage showing on the top of each column.

Of 69 patients, 51 (73.9%) initiated with pure ocular involvement, and the demographic and clinical characteristics of these patients are shown in Table 2. Of the 51 patients with pure ocular onset, 25 (49.0%) underwent secondary generalization. The cumulative survival without generalization was assessed by the Kaplan–Meier method (Figure 2A). Of note, secondary generalization occurred in nearly half of all 25 patients (48.0%) during the first 6 months after onset, 18 (72.0%) patients within 2 years, and 23 (92.0%) within 4 years (Figure 2B).

### Probability of Secondary Generalization of v-LOMG With Pure Ocular Onset

**Figure 3:**
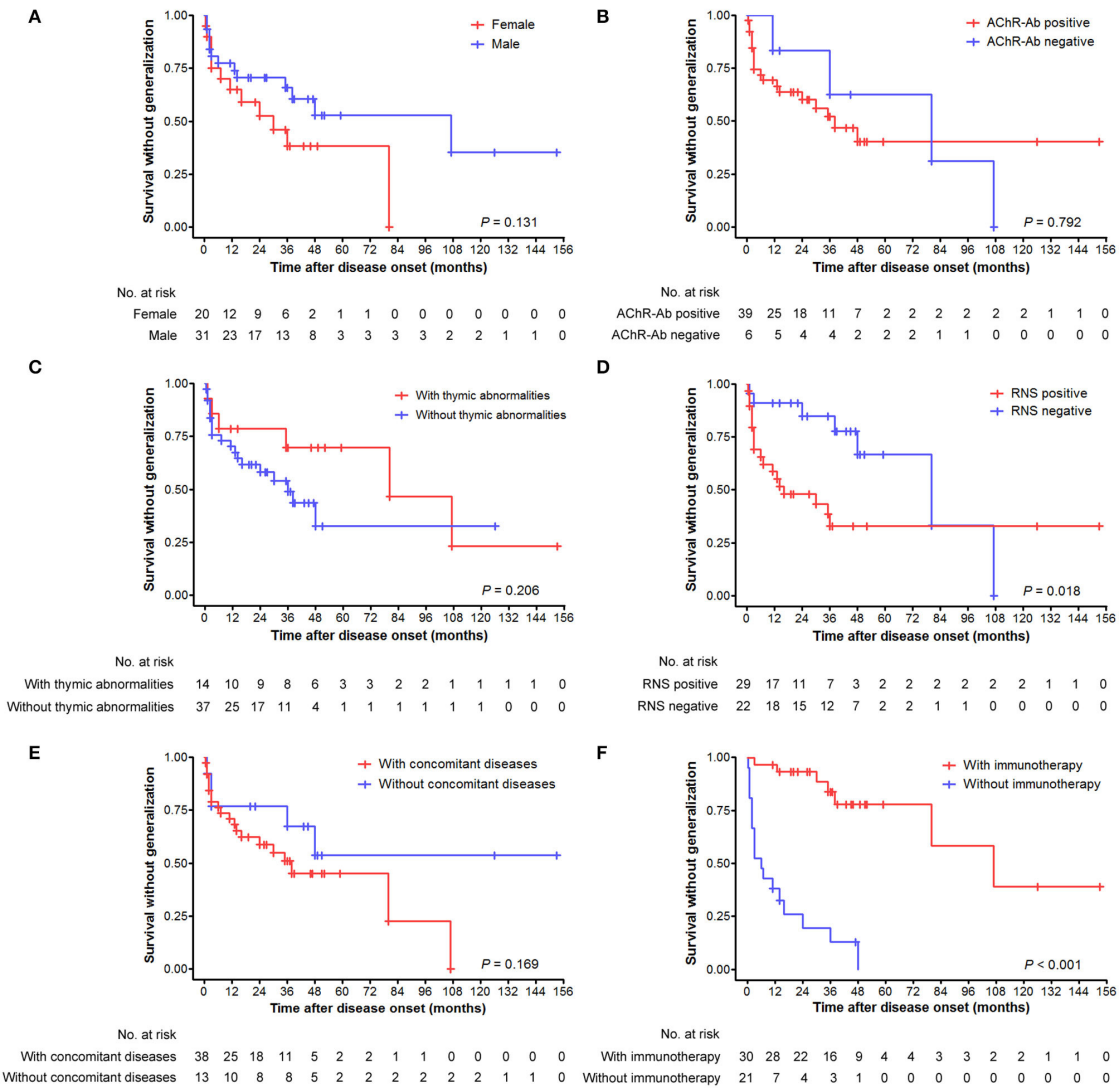
Cumulative survivals without generalization over time (months) as depicted by the Kaplan–Meier curve. **(A)** Comparison between male and female patients. **(B)** Comparison between patients with positive and negative AChR antibody. **(C)** Comparison between patients with and without thymic abnormalities. **(D)** Comparison between patients with positive and negative RNS test results. **(E)** Comparison between patients with and without concomitant diseases. **(F)** Comparison between patients with and without immunotherapy before generalization.

**Figure 4:**
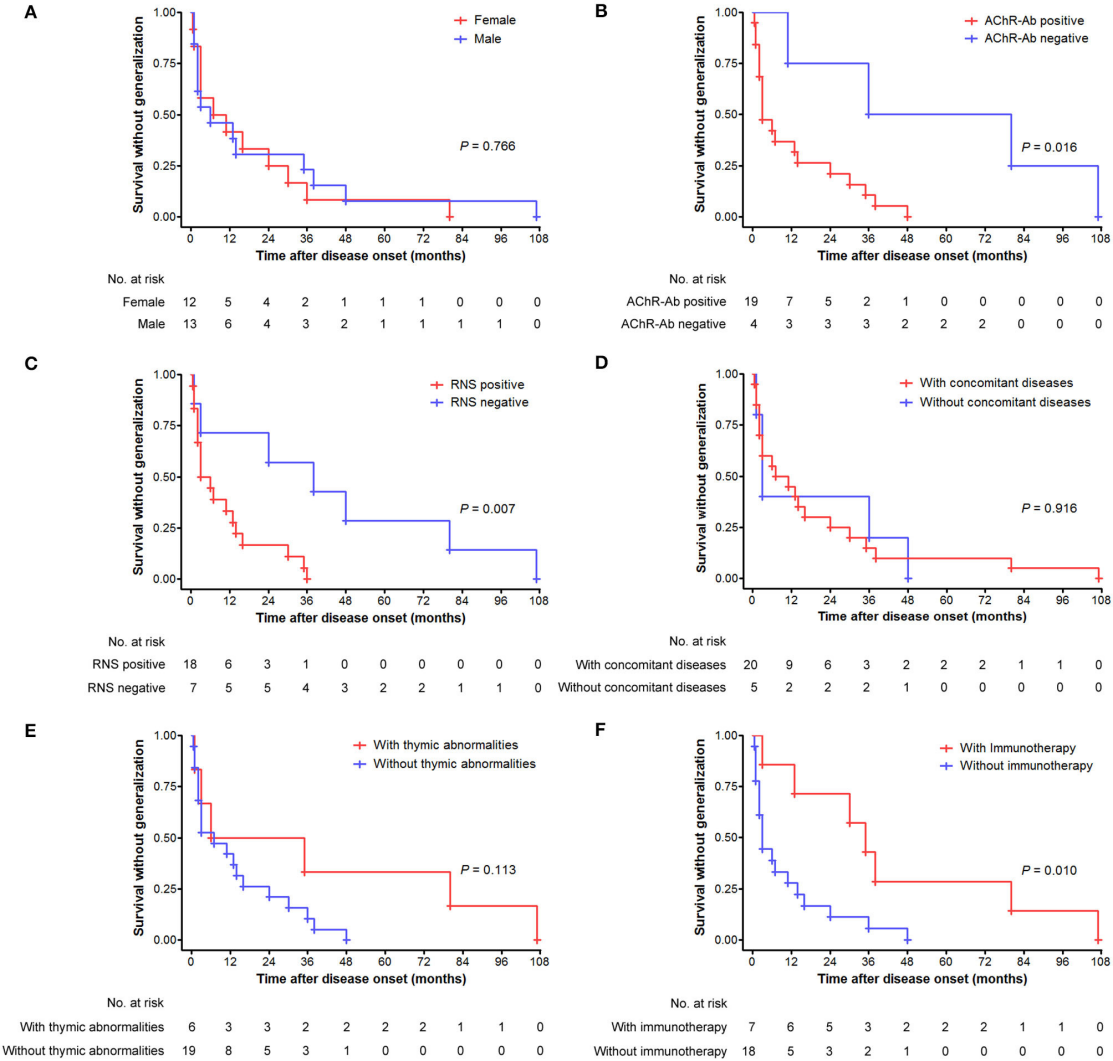
Generalization tempo from disease onset over time (months) as depicted by the Kaplan–Meier curve. **(A)** Comparison between male and female patients. **(B)** Comparison between patients with positive and negative AChR antibody. **(C)** Comparison between patients with positive and negative RNS test results. **(D)** Comparison between patients with and without concomitant diseases. **(E)** Comparison between patients with and without thymic abnormalities. **(F)** Comparison between patients with and without immunotherapy before generalization.

There were no significant differences in the cumulative probabilities of generalization between male and female patients (*p* = 0.131; Figure 3A), those with positive and negative AChR antibody (*p* = 0.792; Figure 3B), those with and without thymic abnormalities (*p* = 0.206; Figure 3C), and those with and without concomitant diseases (*p* = 0.169; Figure 3E), respectively. In contrast, significantly higher probabilities were found in patients with positive RNS results than those with negative results (*p* = 0.018) (Figure 3D). Fifty-one patients with ocular-onset were divided into two groups based on whether or not receiving immunotherapy before generalization and entered into statistical analysis. As revealed in Figure 3F, patients not receiving immunotherapy had a significantly higher probability of generalization than those receiving immunotherapy (*p* < 0.001). We further assessed the intervals from pure ocular onset to generalization in the 25 patients undergoing secondary generalization. Patients with positive AChR antibody, positive RNS results, and not receiving immunotherapy had a shorter time to generalization than those with negative AChR antibody, negative RNS results, and receiving immunotherapy (*p* = 0.016, 0.007, and 0.010, respectively; Figures 4B,C,F), whereas no significant differences were observed between male and female patients (*p* = 0.766; Figure 4A), those with and without concomitant diseases (*p* = 0.916; Figure 4D), and those with and without thymic abnormalities (*p* = 0.113; Figure 4E).

### Predictors of Secondary Generalization in Patients With v-LOMG

Upon Cox regression analysis, acetylcholinesterase inhibitor (AChEI) was excluded because it had been given to all patients. As revealed in Table 3, a total of 10 variables of interest were selected to explore the potential risk factors for secondary generalization. Among these, immunotherapy was the only predictor negatively associated with secondary generalization in patients with v-LOMG with pure ocular onset (HR 0.077, 95%CI [0.024–0.247], *p* < 0.001).

**Table 3:** Cox regression analysis of risk factors for secondary generalization in patients with v-LOMG with pure ocular onset.

**Variables**	**HR**	**95%CI**	***P*-value**
Gender, male vs. female	0.691	0.243–1.962	0.487
Onset age	0.944	0.842–1.058	0.319
Ptosis at onset, unilateral vs. bilateral	1.086	0.235–5.016	0.916
Diplopia at onset, yes vs. no	1.045	0.362–3.017	0.935
AChR antibody, positive vs. negative	1.254	0.226–6.950	0.795
RNS test, positive vs. negative	2.188	0.756–6.332	0.149
Thymic abnormalities, with vs. without	0.778	0.223–2.723	0.695
Concomitant diseases, with vs. without	1.295	0.416–4.027	0.656
Immunotherapy before generalization, with vs. without	0.077	0.024–0.247	<0.001
Time from onset to immunotherapy initiation	0.987	0.971–1.003	0.122

*AChR, acetylcholine receptor; CI, confidential interval; v-LOMG, very-late-onset myasthenia gravis; HR, hazard ratio; RNS, repetitive nerve stimulation*.

## Discussion

In recent years, an accumulating body of studies has demonstrated a true biologic increase in the incidence of elderly onset MG and primarily ascribes this increase to dramatically increased longevity, the aging immune system, and improved diagnostic measures (16–19). Till now, there is a lack of consensus on the definition of this subgroup, and in most studies, the cutoff onset age was defined at 65 or 70 years (7, 19–21). In this study, we included patients with MG with an onset age of 65 years or older and outlined the picture of this subgroup with v-LOMG.

Male predominance is widely recognized in population with elderly onset MG from the Western countries (4, 20–22), whereas female predominance in a Japanese nationwide survey (19), possibly owing to the differences in racial and genetic backgrounds. In this study, a mild male predominance in a small sample of Chinese patients, together with distinct male-to-female ratios in OMG (2.3:1) and GMG (1:1.3) groups further indicate potential gender predominance dependent on clinical subtypes (23). Previous studies enrolling elderly onset MG showed a high prevalence of AChR antibodies ranging from 80% to nearly 93% (4, 6, 19, 21). Similarly, our study showed a comparable positive rate of 85.7%. However, inconsistent with the reported positive rate of 30–77% in the entire OMG population (24), the higher rate of 90.9% in our ocular v-LOMG cohort suggests the role of aging-related changes in the strength of immune response on the differences. Of note, the higher prevalence of AChR antibody in patients with ocular v-LOMG might imply a tendency of secondary generalization in the future. Besides, a higher proportion of GMG than OMG was observed in our cohort. Although this finding is consistent with those from other patient cohorts (4, 19, 21), we cannot completely eliminate the possibility of underdiagnosis of OMG as a result of the ignorance of subtle ocular deficits at the early stage of disease by patients themselves and by clinicians (25).

It is generally accepted that older onset age, positive AChR antibody, the concurrence of thymoma, the use of AChEI, immunotherapy, and smoking can predict secondary generalization of patients with MG with pure ocular onset (3, 8, 26). However, it remains unclear whether such elements have the same effects in the v-LOMG subgroup, given the existence of age-related changes in immune intolerance (27). In this study, Cox regression analysis revealed that immunotherapy was the only predictor negatively associated with secondary generalization, reflecting the most crucial role of immunotherapy in improving the prognosis of v-LOMG. Of particular concern is that, as a specific parameter of elderly patients, concomitant diseases were illustrated not to be associated with a higher probability of secondary generalization. This reflects that no difference in the rate of concomitant diseases was present between patients with pure ocular onset undergoing and not undergoing secondary generalization.

Conclusions drawn from our study and other retrospective studies (9, 18, 21) supported the benefit of immunotherapy in treating patients with v-LOMG; however, in the process of achieving a good prognosis, clinicians often face decision-making difficulties and potential risks. Although our study revealed concomitant diseases were not associated with secondary generalization in patients with v-LOMG, this remains a major consideration in choosing the appropriate treatment strategy. Clinicians tend to be reluctant to treat patients with v-LOMG with an aggressive therapeutic protocol. For elderly patients receiving various medications for comorbidities, added-on immunotherapy might bring undesirable pharmacokinetic and pharmacodynamic drug interactions (28). The fragile and declining immunocompetence in the elderly potentially worsens the situation with the rise of drug-related complications and even leads to increased mortality risk (24, 29, 30). Despite these concerns, the satisfactory efficacy of immunotherapy in v-LOMG subgroup has been reported by several studies (31–33), and the treatment strategy of combining AChEI and rapid immunosuppression followed by chronic immunosuppression was recommended (9). This view was supported by our study where 71.4% of patients treated with AChEI plus immunotherapy achieved favorable outcomes. Furthermore, of 5 patients undergoing myasthenic crisis, only one had been treated with ISs accounting for 1.6% of all patients receiving immunotherapy, in contrast to other 4 patients not receiving immunotherapy (66.7%, *p* < 0.001), indicating the essential role of immunotherapy in preventing serious adverse consequences. Interestingly, there were no significant differences in the percentage of favorable outcomes amongst the steroids alone, immunosuppressant alone, and combined treatment groups. The predictive value of time from onset to immunotherapy initiation was not demonstrated on Cox regression analysis. These findings might be attributed to the small size of our patient cohort, the diversity of immunotherapy selection, and the feasibility of individualized treatment based on the benefit–risk assessment in a real-world setting. Even though the limitations are present, our observation that 80% of myasthenic crises had occurred before the initiation of immunotherapy still highlights the importance of immunosuppression as early as possible. Meanwhile, serious complications associated with immunotherapy cannot be ignored in the elderly, and prophylaxis against side effects of medications should be used to minimize the potential risks.

There are several limitations in this study. First, there was a lack of a unified schedule regarding the coverage of examinations and the timing of follow-up visits, given the nature of this retrospective cohort study. As a result, 8 patients were excluded due to incomplete medical records, and other 9 patients were lost to follow-up. The high exclusion rate of approximately 20% will inevitably affect the strength of our conclusion to some extent. Second, this study included 69 patients with v-LOMG from a single center and only 6 patients did not receive immunotherapy over the course of disease. Meanwhile, there was a lack of muscle-specific kinase antibody-associated MG (MuSK-MG) subgroup that may present distinct clinical features and responses to immunotherapy. The small number and single origin of patients may limit the significance of our conclusion and its scope of application. Third, Cox regression analysis revealed that positive RNS results were close to the borderline level of statistical significance. Considering the recognized predictive value of this variable on secondary generalization in prior studies (11, 26), our finding from v-LOMG subgroup requires further confirmation. Therefore, multicenter, prospective studies involving a larger sample of patients with v-LOMG originated from a wider geographical area are needed in future.

In conclusion, compared to patients with very-late-onset GMG, the counterparts with OMG exhibit a significantly higher female predominance and a lower positive rate of RNS tests, especially on facial and accessory nerves. Notably, lack of immunotherapy is the only predictor of secondary generalization in those patients with v-LOMG with pure ocular onset.

## Data Availability Statement

The raw data supporting the conclusions of this article will be made available by the authors, without undue reservation.

## Ethics Statement

The studies involving human participants were reviewed and approved by Ethics Committees of Tangdu Hospital, Air Force Medical University (Approval Number: TDLL-KY-202105-04). Written informed consent for participation was not required for this study in accordance with the national legislation and the institutional requirements.

## Author Contributions

SZ, KZ, and JG: conceptualization. XY, JD, KR, JL, and CZ: data curation. SS: formal analysis. JG: funding acquisition, project administration, and writing—review and editing. SZ and XY: investigation. SZ, XY, KZ, and JG: methodology. JD, KR, and JL: resources. ZL: supervision. KZ: validation. SZ, XY, and CZ: visualization. SZ: writing—original draft. All authors contributed to the article and approved the submitted version.

## Funding

This study was supported by the Science and Technology Innovation and Development Foundation of Tangdu Hospital (Grant Number 2019LCYJ010) and the Excellent Personnel Foundation of Tangdu Hospital in 2021.

## Conflict of Interest

The authors declare that the research was conducted in the absence of any commercial or financial relationships that could be construed as a potential conflict of interest.

## Publisher's Note

All claims expressed in this article are solely those of the authors and do not necessarily represent those of their affiliated organizations, or those of the publisher, the editors and the reviewers. Any product that may be evaluated in this article, or claim that may be made by its manufacturer, is not guaranteed or endorsed by the publisher.
