# Supplementary material for: Lack of Immunotherapy as the Only Predictor of Secondary Generalization in Very-Late-Onset Myasthenia Gravis With Pure Ocular Onset
Source: Front Neurol. 2022 Apr 25;13:857402. doi: 10.3389/fneur.2022.857402 (PMC9081806; doi:10.3389/fneur.2022.857402)
Supplement: Supplementary file 1 [file Data_Sheet_1.PDF]

Supplementary Table 1 Comparison of outcomes between v-LOMG patients with/without therapies and different duration of therapy

Outcomes	*w/o* IT	*w* IT	*P* value	Short-term IT	Long-term IT	*P* value
	N = 6	N = 63		N = 11	N = 52	
Favorable, *n* (%)	0 (0)	45 (71.4)	**0.001**	7 (63.6)	38 (73.1)	0.714
Intermediate, *n* (%)	2 (33.3)	10 (15.9)	0.278	2 (18.2)	8 (15.4)	1.000
Unfavorable, *n* (%)	3 (50.0)	3 (4.8)	**0.007**	2 (18.2)	1 (1.9)	0.076
Poor, *n* (%)	1 (16.7)	5 (8.0)	0.433	0 (0)	5 (9.6)	0.576

Abbreviations: v-LOMG, very-late-onset myasthenia gravis; OMG, ocular myasthenia gravis; GMG, generalized myasthenia gravis; *w* IT, with immunotherapy; *w/o* IT, without immunotherapy. Outcomes were evaluated by the Myasthenia Gravis Foundation of America Post-Intervention Status (MGFA-PIS). Favorable outcomes were defined as the achievement of minimal manifestations (MM) or better, including complete stable remission (CSR), pharmacologic remission (PR), and MM. An intermediate outcome was considered as a status of improved (I); unfavorable outcomes as unchanged (U), worse (W), and exacerbation (E); and a poor outcome as died (D) of MG. Long-term IT was defined as the duration of therapy longer than 6 months and short-term IT as shorter than 6 months. Inter-group differences were analyzed by Fisher's exact test.

Supplementary Table 2 Long-term outcomes of 63 v-LOMG patients receiving different treatment regimens

Outcomes	Steroids	Steroids + IS	IS	*P* value
	N = 9	N = 46	N = 8	
Favorable, *n* (%)	6 (66.7)	34 (73.9)	5 (62.5)	0.759
Intermediate, *n* (%)	2 (22.2)	8 (17.4)	0 (0)	0.394
Unfavorable, *n* (%)	0 (0)	2 (4.3)	1 (12.5)	0.467
Poor, *n* (%)	1 (11.1)	2 (4.3)	2 (25.0)	0.127

Abbreviations: v-LOMG, very-late-onset myasthenia gravis; IS, immunosuppressant. Immunosuppressants included azathioprine, tacrolimus, mycophenolate mofetil, or intravenous immunoglobulin (IVIg). Outcomes were evaluated by the Myasthenia Gravis Foundation of America Post-Intervention Status (MGFA-PIS). Favorable outcome was defined as the achievement of minimal manifestations (MM) or better, including complete stable remission (CSR), pharmacologic remission (PR) and MM. Intermediate outcome was considered as a status of improved (I); unfavorable outcome as unchanged (U), worse (W) and exacerbation (E); and poor outcome as died (D) of MG. Inter-group differences were analyzed by χ^2 test.
